# P48 An elusive parasite: a case of babesiosis in an immunocompetent traveller unmasked by a blood smear

**DOI:** 10.1093/jacamr/dlaf230.055

**Published:** 2025-12-04

**Authors:** Melissa Prior-Ong

**Affiliations:** Microbiology Department, Torbay and South Devon Foundation Trust, Lowes Bridge, Torbay Hospital, Torquay TQ2 7AA, UK

## Abstract

**Background:**

Babesiosis is a tick-borne zoonosis caused by protozoa of the *Babesia* genus, most commonly *Babesia microti and Babesia divergens*. Endemic to the Northeastern and Midwestern United States and parts of Europe respectively, infection is primarily transmitted via *Ixodes* tick bites. While often subclinical, especially in immunocompetent individuals, babesiosis can progress to severe disease—including multiorgan dysfunction—in vulnerable populations such as the elderly, asplenic or immunocompromised. Despite its epidemiological footprint, babesiosis remains challenging to diagnose relying on clinical suspicion in febrile retuning travellers. We describe the case of an immunocompetent patient, where the identification of intraerythrocytic trophozoites on a routine peripheral blood smear prompted a re-evaluation of the clinical narrative.

**Case presentation:**

A previously well 70-year-old woman presented to hospital after being found collapsed at home, preceded by a 5 day history of malaise, dizziness and intermittent fevers. On admission, the patient was febrile and reported abdominal pain. She was empirically treated with IV cefuroxime and metronidazole for presumed intra-abdominal sepsis and admitted under the surgical team. Initial laboratory evaluation revealed thrombocytopenia (platelet count 68 ×10⁹/L), normocytic anaemia and elevated inflammatory markers. A routine peripheral blood smear performed for thrombocytopenia unexpectedly demonstrated intraerythrocytic ring forms, with a parasitaemia of 1.3%. Molecular testing via PCR confirmed *Babesia microti*. Notably, the patient had returned three weeks earlier from a trip to the East Coast of the USA, including travel through tick-endemic areas in upstate New York, New Jersey and Pennsylvania. Although she did not recall a tick bite, exposure to a wooded environment and a family member’s tick encounter provided important epidemiological context.

**Discussion:**

This case underscores the diagnostic complexity of babesiosis, particularly in immunocompetent hosts with non-specific symptoms and without a known tick bite. The initial clinical picture—suggestive of bacterial sepsis—delayed consideration of parasitic infection. The diagnostic pivot hinged on the laboratory identification of parasitaemia, highlighting the indispensable role of peripheral blood smear review in haematological abnormalities. Early recognition enabled prompt initiation of targeted therapy with IV clindamycin and oral quinine, followed by transfer to a tertiary centre for continued management including non-invasive ventilation and red blood cell transfusion. She subsequently made a full recovery.

**Conclusions:**

This case exemplifies the diagnostic interplay between clinical acumen and laboratory vigilance in identifying imported vector-borne infections. It also reinforces the need to consider babesiosis in the differential diagnosis of febrile illness with cytopenias in returning travellers from endemic regions—even in the absence of classic risk factors or tick exposure history. The timely detection of *Babesia* on blood smear was central to altering the diagnostic trajectory and facilitating effective antimicrobial therapy.

